# Overexpression of *TCP8* delays Arabidopsis flowering through a *FLOWERING LOCUS C*-dependent pathway

**DOI:** 10.1186/s12870-019-2157-4

**Published:** 2019-12-03

**Authors:** Xiaoyan Wang, Xintong Xu, Xiaowei Mo, Luyao Zhong, Jiancong Zhang, Beixin Mo, Benke Kuai

**Affiliations:** 1 Guangdong Provincial Key Laboratory for Plant Epigenetics, College of Life Sciences and Oceanography, Shenzhen University, Shenzhen, 518060 China; 2 State Key Laboratory of Genetic Engineering, School of Life Sciences, Fudan University, Shanghai, 200438 China

**Keywords:** TCP8, Flowering, FLC, Vernalization, Arabidopsis

## Abstract

**Background:**

Flowering is a key process in the life cycle of plants. The transition from vegetative to reproductive growth is thus under sophisticated regulation by endogenous and environmental signals. The plant-specific Teosinte Branched 1/Cycloidea/Proliferating Cell Factors (TCP) family transcription factors are involved in many biological processes, but their roles in regulating flowering have not been totally elucidated.

**Results:**

We explored the role of Arabidopsis *TCP8* in plant development and, especially, in flowering control. Overexpression of *TCP8* significantly delayed flowering under both long-day and short-day conditions and dominant repression by *TCP8* led to various growth defects. The upregulation of *TCP8* led to more accumulated mRNA level of *FLOWERING LOCUS C* (*FLC*), a central floral repressor of Arabidopsis. *TCP8* functions in an FLC-dependent manner, as *TCP8* overexpression in the *flc-6* loss-of-function mutant failed to delay flowering. The vernalization treatment could reverse the late flowering phenotype caused by *TCP8* overexpression.

**Conclusions:**

Our results provide evidence for a role of *TCP8* in flowering control and add to our knowledge of the molecular basis of *TCP8* function.

## Background

The transition from vegetative to reproductive growth is one of the most important processes in the life cycle of flowering plants. As a result, flowering is under strict and sophisticated regulation by multiple endogenous cues and environmental signals [1]. Many studies using the model plant *Arabidopsis thaliana* (*A. thaliana*) have identified major flowering-related genes, these genes were found to be involved in photoperiod, vernalization, gibberellin, aging, temperature, and the autonomous pathways [2–4]. For example, the central floral repressor *FLOWERING LOCUS C* (*FLC*) is involved in both the vernalization and the autonomous pathways. During vernalization, FRIGIDA (FRI) activates *FLC* expression while prolonged cold repressed *FLC* expression [5, 6]. In the autonomous pathways, *FLOWERING CONTROL LOCUS A* (*FCA*), *FLOWERING LOCUS PA* (*FPA*), *FLOWERING LOCUS KH DOMAIN* (*FLK*), *FLOWERING LOCUS Y* (*FY*), *LUMINIDEPENDENS* (*LD*), *FLOWERING LOCUS VE* (*FVE*), and *FLOWERING LOCUS D* (*FLD*) repress *FLC* expression under both long-day (LD) and short-day (SD) conditions [7, 8]. The MADS-box transcription factor FLC directly binds to the chromatin of floral integrator *FLOWERING LOCUS T* (*FT*) and *SUPPRESSOR OF OVEREXPRESSION OF CONSTANS 1* (*SOC1*) to repress flowering [9, 10]. *FLC* acts as a central floral repressor by converging the signal from different pathways in a dose-dependent manner [11]. Its expression is under sophisticated control at both transcriptional and post-transcriptional levels by diverse regulate factors [12–14]. Through massive studies, the *FLC* locus not only shows the delicate regulation involving flowering control, but also provides an important platform for discovering epigenetic regulation of gene expression [14].

Members of the Teosinte Branched 1/Cycloidea/Proliferating Cell Factors (TCP) family are plant-specific transcription factors involved in many biological processes including flowering [15]. The Arabidopsis *TCP* family consists of 24 genes and can be further divided into two clades, class I and class II, based on their sequence features [16]. Involvement of the TCPs from both classes in regulating flowering has been reported in previous studies. For example, TCP15 promotes flowering by directly regulating *SOC1* expression [17], and TCP4 functions as a transcriptional activator by directly binding to the *CONSTANS* (*CO*) promoter to induce flowering [18, 19]. Several TCPs can regulate floral transition by interacting with FT through protein-protein interaction [20, 21]. TCPs also affected flowering through circadian –related pathways. For example, TCP20 and TCP22 are positive regulators of *CIRCADIAN CLOCK ASSOCIATED 1* (*CCA1*), while TCP21 represses *CCA1* expression [22, 23]. Despite the importance, the molecular mechanism underlying the TCP-FLC interaction remains to be elucidated.

Our previous work demonstrated TCP8, a member of class I TCPs, directly binds to the *ISOCHORISMATE SYNTHASE 1* promoter to activate plant immune response [24]. Besides, TCP8 also binds and activates promoter of *EF-TU RECEPTOR* in planta during pathogenesis [25]. In addition, TCP8 interacts with NON-EXPRESSER of PR GENES 1 and SUPPRESSOR OF rps4-RLD1 via protein-protein interaction after pathogen attack [26, 27]. These results clearly showed a role of TCP8 participating in plant-pathogen interaction. Other studies have also suggested TCP8 may have roles during plant development and yield determination [28–33], but its function in regulating flowering time is not clear. In this study, we investigate the role of *TCP8* in flowering control by overexpression. We found that *TCP8* overexpression delays plant flowering in an FLC-dependent manner. Furthermore, TCP8 and its homologs are indispensable for plant development.

## Results

### *TCP8* is ubiquitously expressed during plant development

**Fig. 1:**
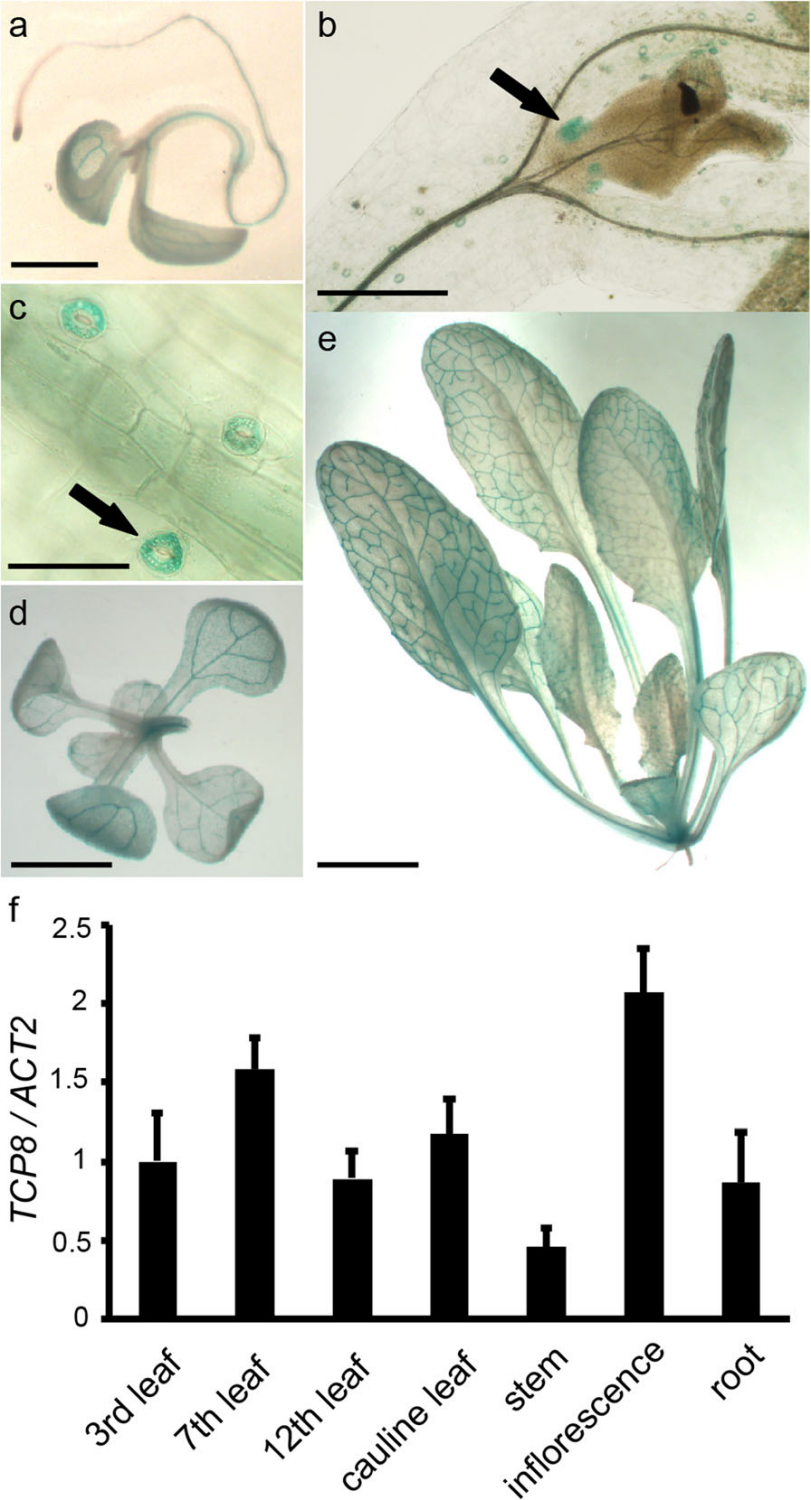
*TCP8* is ubiquitously expressed throughout the plant during development. **a-e.** Histochemical staining of the *pTCP8::GUS* lines for GUS activity. a-c. GUS staining of five-day-old *pTCP8::GUS* seedlings, the black arrows indicate leaf primordial (**b**) and stomatal guard cell (**c**). **d** and **e**. Twelve-day-old and 20-day-old *pTCP8::GUS* seedlings. **f.** Relative transcription levels of *TCP8* in different tissues detected by RT-qPCR. Data are represented as mean ± standard deviation (S.D.) of three biological replicates. *ACTIN2* was used as the endogenous control for normalizing the relative transcription levels of *TCP8*. The transcription level of *TCP8* relative to *ACTIN2* in the 3rd-leaf was arbitrarily set to 1. The scale bars are 20 μm (**a**), 10 μm (**b**), 4 μm (**c**), 5 mm (**d**), and 1 cm (**e**), respectively

To characterize the tissue-specific expression pattern of *TCP8* at different developmental stages in detail, a 1.5 kb promoter region upstream of the start codon of *TCP8* was fused with the *GUS* gene and the construct was transformed into Columbia-0 (Col-0). Several independent transgenic lines exhibited similar patterns of GUS expression. *TCP8* showed a ubiquitous expression pattern in the transgenic plants—GUS signal was mainly detected in the vascular bundles in cotyledons, primary roots, hypocotyls and rosette leaves throughout development (Fig. 1a, d, e). Relatively higher *TCP8* expression was detected in leaf primordia and stomatal guard cells (Fig. 1b, c), suggesting a potential role of *TCP8* in tissue initiation and stomatal function. We then validated the histochemical GUS staining results by real-time quantitative PCR (RT-qPCR). Consistently, *TCP8* expression was detected in all the tissues tested—including rosette leaves, cauline leaves, stem, inflorescence and root (Fig. 1f). Taken together, these results revealed the ubiquitous expression pattern of *TCP8* throughout development and a potentially function in flowering.

### *TCP8* overexpression delayed flowering under both LD and SD conditions

**Fig. 2:**
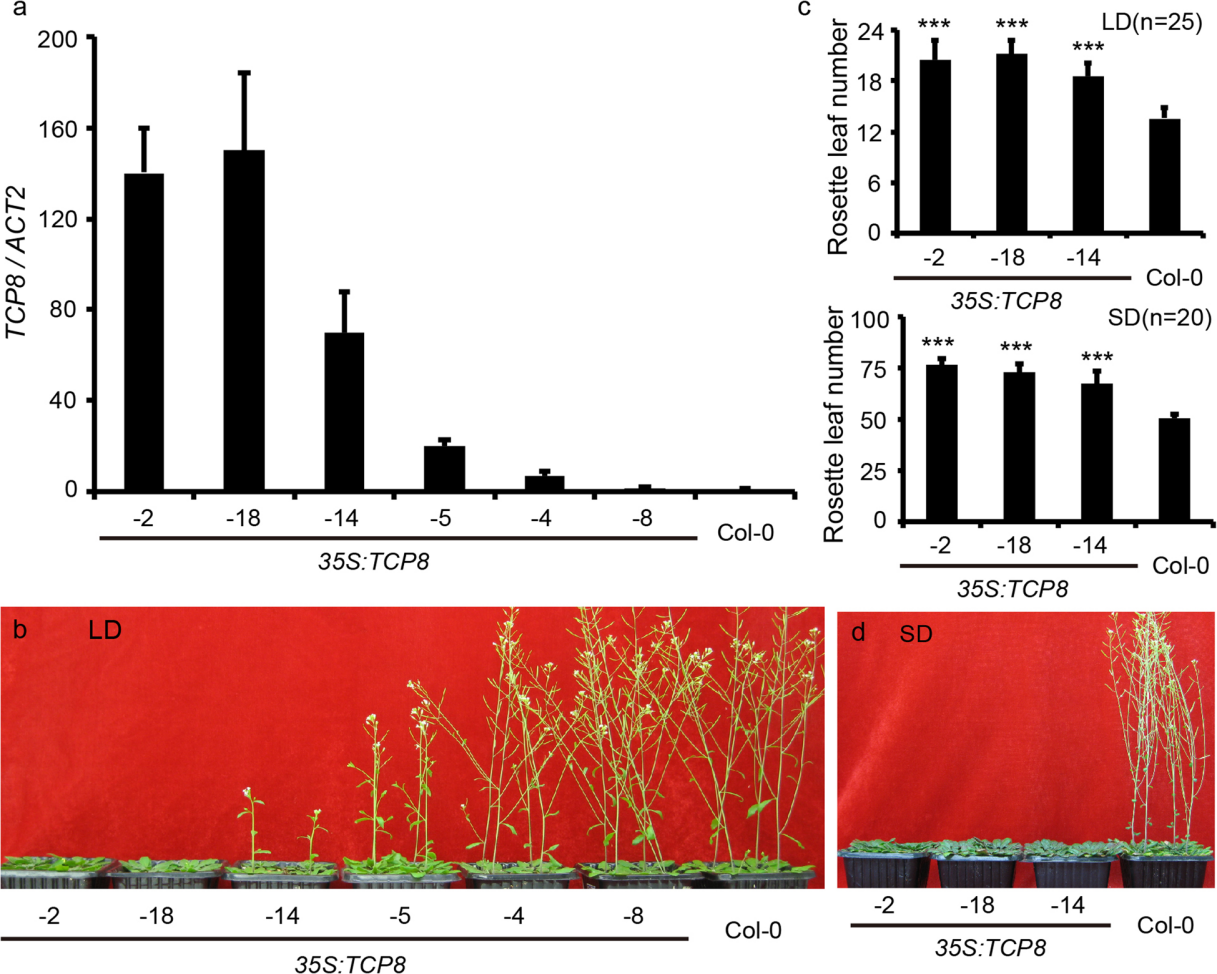
Overexpression of *TCP8* delays flowering. **a**. Relative transcription levels of *TCP8* in different *35S::TCP8* transgenic lines detected by RT-qPCR. Data are represented as mean ± SD of three biological replicates. *ACTIN2* was used as the endogenous control for normalizing the transcription levels of *TCP8*. The transcription level of *TCP8* in Col-0 was arbitrarily set to 1. **b.** Forty five-day-old Col-0 and *35S:TCP8* transgenic plants grown under LD condition. **c.** The number of rosette leaves in wild-type Col-0 and *35S::TCP8* plants before bolting (Student’s *t*-test: ***P < 0.001). **d.** Seventy-day-old wild-type Col-0 and *35S::TCP8* transgenic plants grown under the SD condition

To further investigate the role of *TCP8*, we overexpressed *TCP8* using the constitutive *35S cauliflower mosaic virus* promoter (*35S*) in Col-0 and homozygous T3 progeny were analyzed. The *35S::TCP8* plants showed a clear late-flowering phenotype compared with wild-type (WT) Col-0 under the long-day (LD) condition, and the extent of delay well correlated with the relative transcription levels of *TCP8* (Fig. 2a, b). A similar late-flowering phenotype was also observed for the *35S::TCP8* plants under the short-day (SD) condition (Fig. 2d). It is well accepted that late-flowering plants often generate more rosette leaves before flowering. Indeed, the *TCP8* overexpression plants generated more rosette leaves than control plants both under LD and SD (Fig. 2c), demonstrating that TCP8 is a bona fide regulator of Arabidopsis flowering. Moreover, we observed retarded growth with the *35S::TCP8* individuals, although the final plant height of *35S::TCP8* were comparable to those of the WT (Additional file 1: Figure S1).

Next, we expressed *TCP8* in Col-0 with the native promoter of *TCP8* to eliminate the effect of ectopically expressed *TCP8* on flowering. The *pTCP8::TCP8* plants phenocopied *35S::TCP8* plants by showing a delayed flowering compared with the WT control (Additional file 2: Figure S2), although the late-flowering phenotype was less obvious compared with the *35S::TCP8* individuals, probably due to a lower expression levels of *TCP8* under its native promoter. Collectively, these data provide evidence for a role of *TCP8* in regulating plant flowering.

### Overexpression of *TCP8* up-regulates *FLC* mRNA level

**Fig. 3:**
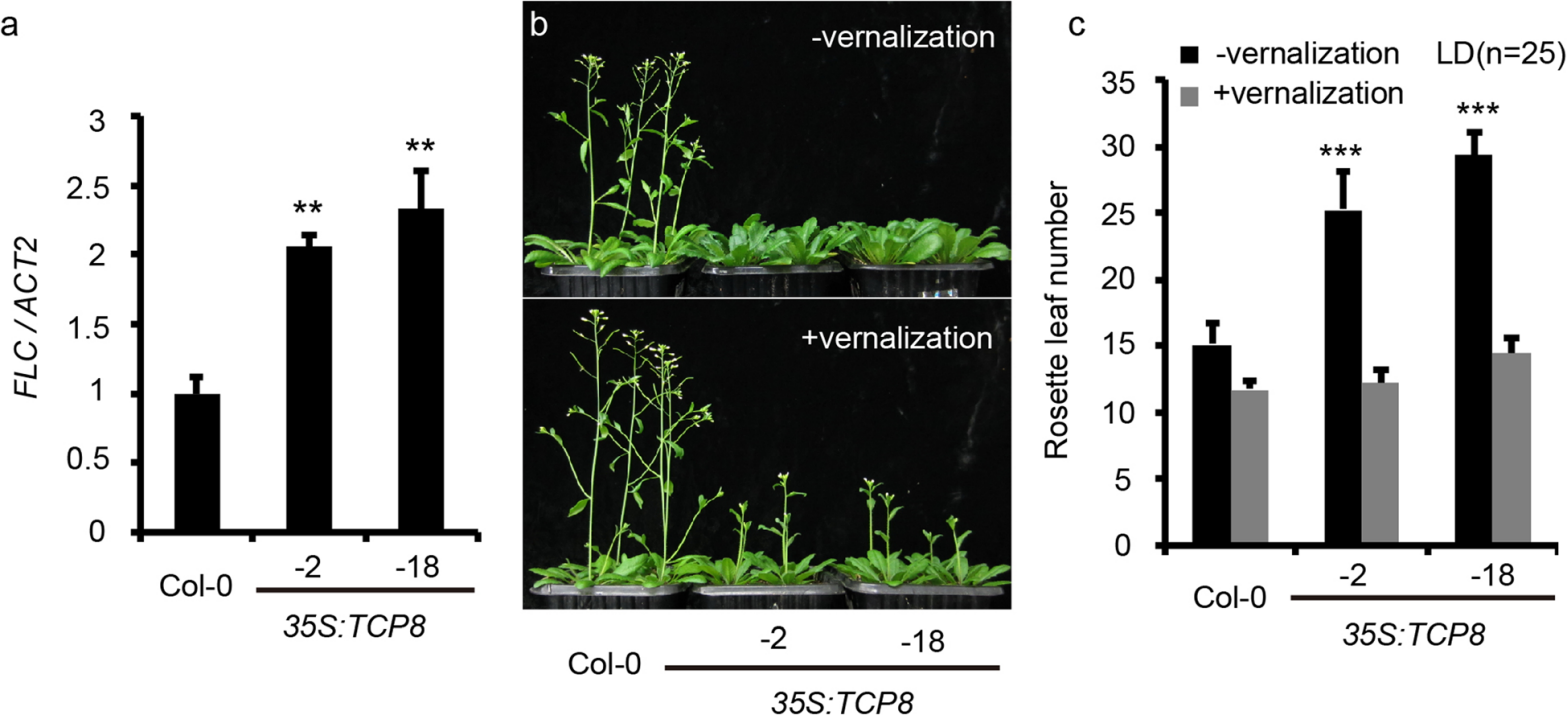
Overexpression of *TCP8* up-regulates *FLC* mRNA level. **a.** Relative transcription levels of *FLC* in *35S::TCP8* seedlings detected by RT-qPCR. Data are shown as mean ± SD of three biological replicates. *ACTIN2* was used as the endogenous control for normalizing the transcription levels of *FLC*. The transcription level of *FLC* in wild-type Col-0 was arbitrarily set to 1. (Student’s *t* test: **P < 0.01). **b.** Forty-day-old wild-type Col-0 and *35S::TCP8* plants grown under LD condition with or without vernalization. **c.** The number of rosette leaves in wild-type Col-0 and *35S::TCP8* plants before bolting. (Student’s *t*-test, ** *P* < 0.01, ***P < 0.001)

The delayed flowering phenotype of *35S::TCP8* plants under both LD and SD conditions suggest a putative role of *TCP8* in the autonomous pathways. Thus, we examined the transcription levels of *FLC—*a master repressor of flowering in the autonomous pathways*—*in the *35S::TCP8* lines. Consistent with our hypothesis, *FLC* transcription level in the *35S::TCP8* lines increased by over two folds compared with WT control during early developmental stages (Fig. 3a). This result suggests that *TCP8* may control flowering by upregulating *FLC* mRNA level.

Vernalization has been shown as an effective way to reduce native *FLC* expression, so we checked whether vernalization could rescue the late-flowering phenotype of *TCP8* overexpression plants. As expected, vernalization greatly rescued the delayed flowering of *TCP8* overexpression plants (Fig. 3b). We observed no obvious difference in the number of roseate leaves between the *35S::TCP8* and control plants in the vernalization group (Fig. 3c), confirming a potential role of *FLC* in *TCP8*-mediated flowering control. It is of interest to note that the number of days after germination for flowering of *TCP8* overexpression plants were still more than WT, presumably owing to the retarded growth caused by *TCP8* overexpression (Fig. 3b). That is, vernalization specifically rescued the late-flowering phenotype caused by *TCP8* overexpression, but had no effect on its growth hindering effect.

To further investigate the molecular mechanism of *TCP8* in controlling flowering, we tested the binding of TCP8 to *FLC* promoter in *TCP8-GFP* transgenic plants [24] using the chromatin immunoprecipitation (ChIP) assay and the in vitro electrophoretic mobility shift assay (EMSA) (Additional file 3: Figure S3). Neither the ChIP assay nor the EMSA detected an interaction between TCP8 and the *FLC* promoter, suggesting that TCP8 indirectly regulated *FLC* expression. Considering the Col-0 ecotype contains a *fri*-null allele, TCP8 may regulate genes upstream of FLC in the autonomous pathway, which were thought to cooperatively repress *FLC* expression. Therefore, we measured the relative expression levels of *FCA*, *FPA*, *FLK*, *FY*, *LD*, *FVE*, and *FLD* in the *TCP8* overexpression seedlings and observed moderate decreases in *FCA*, *FLK*, *LD* and *FLD* expression (Additional file 4: Figure S4a). In addition, a group of antisense long noncoding transcripts termed *COOLAIR* are reported to regulate *FLC* expression level [34]. But we did not detect a significant change of *COOLAIR* levels in *TCP8* overexpression seedlings (Additional file 4: Figure S4b). Taken together, these analyses revealed that *TCP8* overexpression indirectly up-regulted *FLC* mRNA level, probably through the down-regulation of a set of autonomous genes.

### FLC is required for *TCP8*-mediated flowering control

**Fig. 4:**
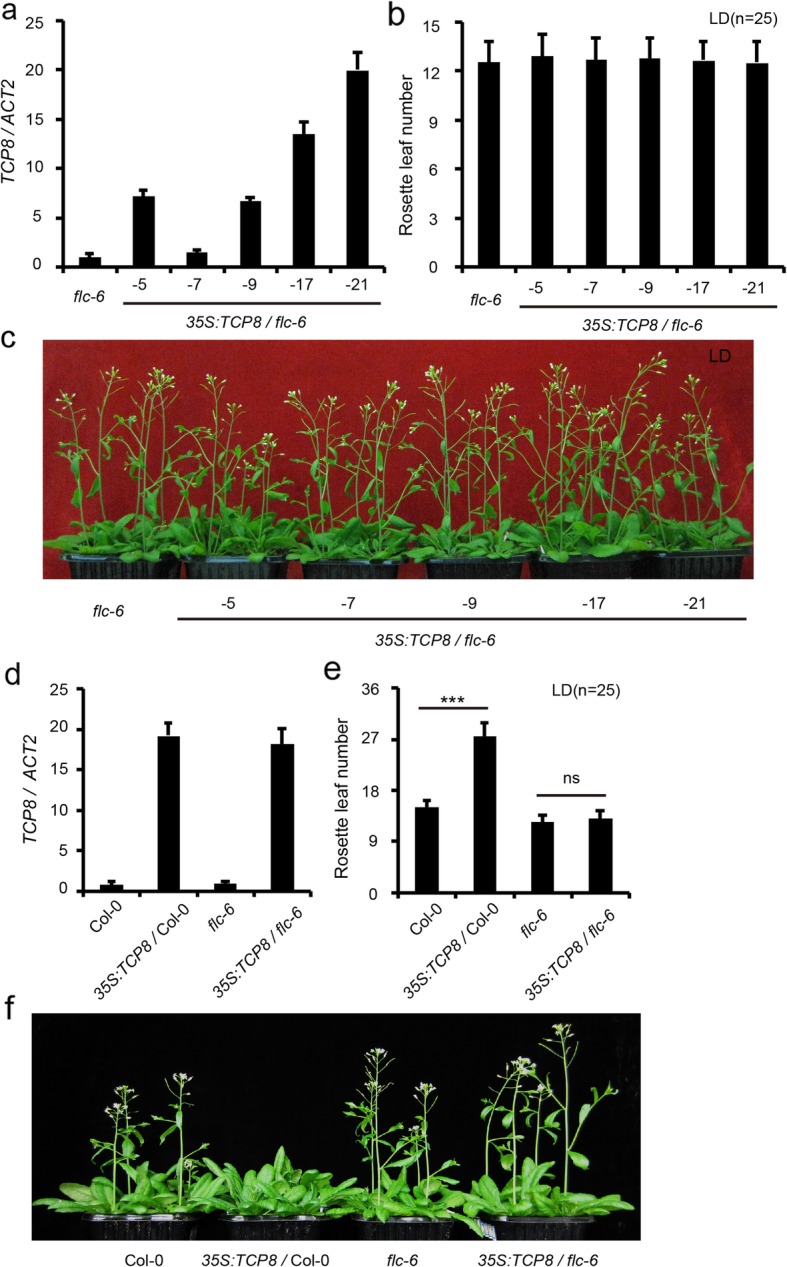
*FLC* is required for the TCP8 function in regulating flowering time. **a.** Relative transcription levels of *TCP8* in *flc-6* and *35S::TCP8/flc-6* detected by RT-qPCR. Data are represented as mean ± S.D. of three biological replicates. *ACTIN2* was used as the endogenous control for normalizing the transcription levels of *TCP8*. The transcription level of *TCP8* in *flc-6* was arbitrarily set to 1. **b.** The number of rosette leaves in *flc-6* and *35S::TCP8/flc-6* plants before bolting. **c.** Forty-day-old *flc-6* and *35S::TCP8/flc-6* plants grown under LD condition. **d.** Transcription levels of *TCP8* in wild-type Col-0, *35S::TCP8*, *flc-6* and *35S::TCP8/flc-6* individuals. *ACTIN2* was used as the endogenous control for data normalization and the transcription level of *TCP8* is represented as mean ± S.D. of three biological replicates. The transcription level of *TCP8* in Col-0 was arbitrarily set to 1. **e.** The number of rosette leaves in wild-type Col-0, *35S::TCP8*, *flc-6* and *35S::TCP8/flc-6* before bolting. (Student’s *t*-test, *** *P* < 0.001, ns = not significant). **f.** Forty-day-old wild-type Col-0, *35S::TCP8*, *flc-6* and *35S::TCP8/flc-6* plants grown under LD condition

To validate the role of FLC in TCP8-regulated flowering, we overexpressed *TCP8* in an *FLC* loss-of-function mutant, *flc-6*. *TCP8* overexpression had no obvious effect on the flowering of *flc-6*—no significant difference in rosette leaves number was observed between the *flc-6* and *35S::TCP8/flc-6* individuals—despite high expression of *TCP8* was detected in *35S::TCP8/flc-6* plants (Fig. 4a-c). We then crossed the *35S::TCP8* transformants in Col-0 with *flc-6*. In the F2 segregation population, *TCP8* overexpression in *flc-6* background showed comparative flowering time with *flc-6*, while *TCP8* overexpression in *FLC* WT background showed significant delayed flowering (Fig. 4d-f), suggesting that a functional *FLC* gene is required for *TCP8* function. We obtained similar results in *pTCP8:TCP8* and *flc-6* crossed F2 progeny (Additional file 5: Figure S5). Therefore, *FLC* is essential for the *TCP8-*mediated flowering control.

### Dominant repression by *TCP8* leads to various growth defects

**Fig. 5:**
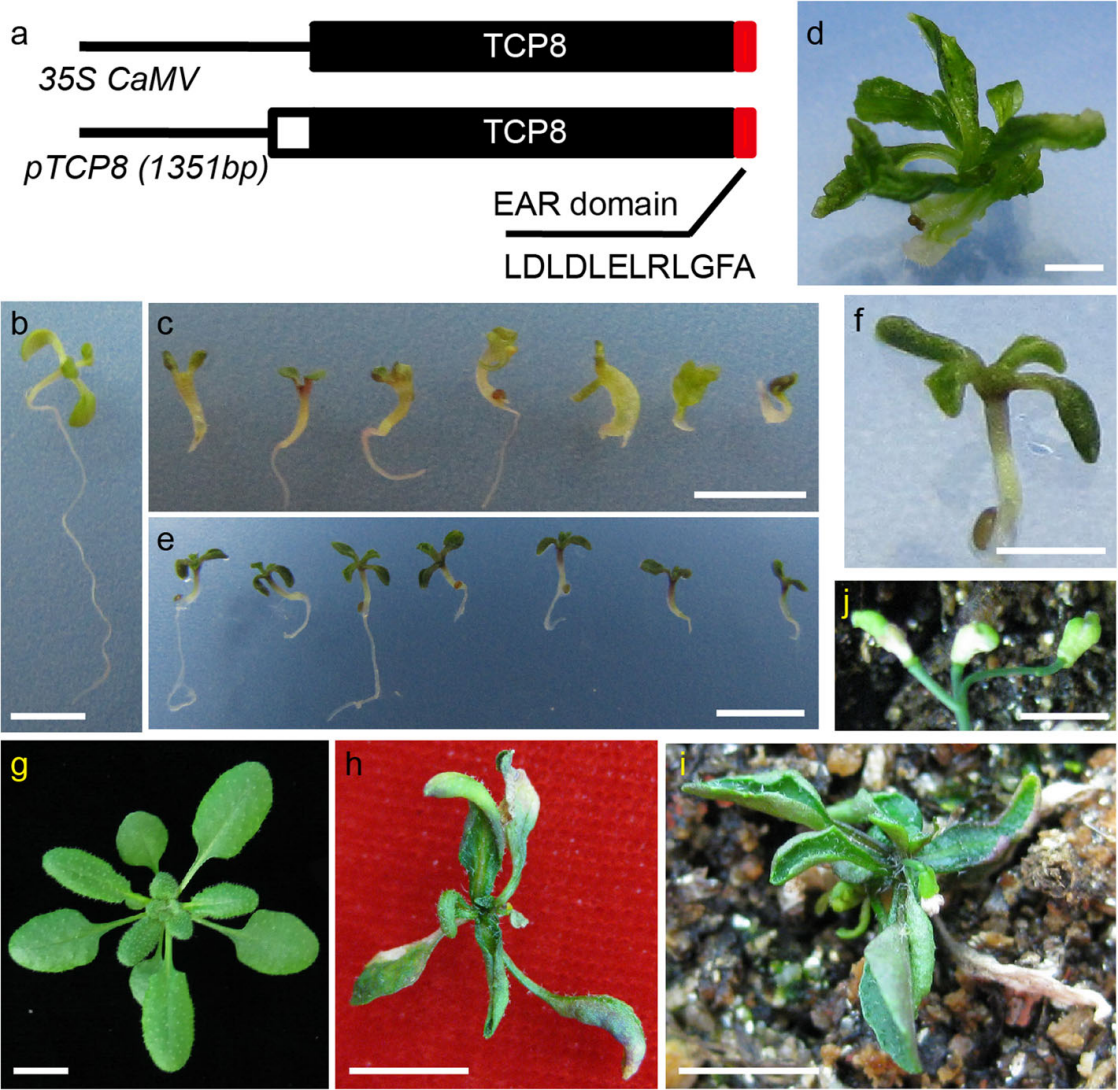
Phenotypic characterization of the TCP8-EAR transgenic plants. **a.** Schematic representation of the *35S::TCP8* and *pTCP8::TCP8-EAR* constructs. Black box represents the open reading frame of *TCP8*, red box represents the EAR motif consisting of 12 amino acids, and white box represents the 5′ untranslated region of *TCP8*. **b.** Seven-day-old wild-type Col-0 seedling. **c.** Seven-day-old *35S:TCP8-EAR* seedlings. **d** A twenty-day-old *35S:TCP8-EAR* plant grown on half-strength MS plate. **e** and **f.** Seven-day-old *pTCP8::TCP8-EAR* seedlings. **g.** A twenty five-day-old wild-type Col-0 plant. **h.** A twenty five-day-old *pTCP8::TCP8-EAR* plant. **i. A** *pTCP8::TCP8-EAR* plant in the reproductive stage. **J.** The inflorescence of *pTCP8::TCP8-EAR*. The scale bars are 5 mm (b-e), 2.5 mm (f), 1 cm (g-i), and 5 mm (j), respectively

A *TCP8* mutant generated by T-DNA insertion (*tcp8–1*, *CS875709*) was obtained from ABRC to investigate the role of *TCP8* in flowering. However, we observed no visible defect in flowering and development (Additional file 6: Figure S6), which may be owing to the functional redundancy among TCP family members, as proposed in previous studies [35]. To overcome this redundancy, we fused the EAR motif to the C-terminus of TCP8 and overexpressed the TCP8-EAR fusion protein in Col-0 to mediate dominant repression by *TCP8* (Fig. 5a). The *35S::TCP8-EAR* plants exhibited severe developmental defects—the growth of most plants was arrested at the seedling stage (Fig. 5c). The survived *35S:TCP8-EAR* seedlings failed to develop normal leaves (Fig. 5d). Similarly, we observed a moderate level of growth defect with the *pTCP8::TCP8-EAR* seedlings, which had dark green leaves, hyponastic cotyledons, and shorter primary roots (Fig. 5e, f); some *pTCP8::TCP8-EAR* seedlings could develop curved true leaves but few made to the reproductive stage (Fig. 5h). The adult plants of *pTCP8::TCP8-EAR* were considerably smaller than Col-0 and could not develop a normal inflorescence—the three outer whorls of the *pTCP8::TCP8-EAR* flower were fused and the irregular gynoecia was exposed (Fig. 5i, j). The *pTCP8::TCP8-EAR* plants also failed to develop viable seeds. Taken together, these results point to an indispensable role of *TCP8* in maintaining normal plant growth and flower development.

## Discussion

Functional redundancy often hinders the analysis of the TCP family members [15]. For example, *TCP8*, *TCP14*, and *TCP15* are functionally redundant in regulating development and immune response [25–28, 30], however, *TCP8* and *TCP15* may play different roles in controlling flowering. Overexpression of *TCP15* promoted early flowering and the *tcp15* loss-of-function mutant showed delayed flowering [17]. In the present study, the *tcp8* single mutant had no obvious phenotype but *TCP8* overexpression led to delayed flowering (Fig. 2b-d). According to previous studies, TCP15 directly activates *SOC1* expression [17], and TCP8 functions upstream of *FLC* (Fig. 4), suggesting that TCP8 functions upstream of *SOC1*. In contrast to that observed with the immune response, *TCP8* and *TCP15* may have no redundancy in flowering regulation. *TCP23* overexpression could also delay flowering under LD condition [36], suggesting that *TCP23* and *TCP8* may have overlapping functions in flowering control. To overcome the functional redundancy among TCP members, the chimeric TCP8-EAR protein was expressed in WT Col-0 to introduce dominant *TCP8* repression, which resulted in various growth defects in the transgenic plants (Fig. 5). Compared with *TCP14* and *TCP15* dominant repression [37–39], the *TCP8-EAR* plants exhibited more severe developmental defects. This observation is consistent with the ubiquitous expression pattern of *TCP8* during seedling development (Fig. 1).

*TCP8* overexpression delayed flowering under both LD and SD conditions and *TCP8* functions upstream of *FLC*, suggesting that *TCP8* may play a role in the autonomous pathways. Consistent with this hypothesis, we found that the expression levels of several known autonomous pathway regulators, including *FCA*, *FLK*, *LD,* and *FLD*, were downregulated in the *TCP8* overexpression lines (Additional file 4: Figure S4a), suggesting that TCP8 may indirectly affect *FLC* expression through regulating these genes. Consistent with this assumption, we failed to detect a direct interaction between TCP8 and the promoter of *FLC* in the EMSA and ChIP assay. Previous studies have suggested that the autonomous pathway factors mainly regulate *FLC* expression via post-transcriptional RNA-processing and epigenetic mechanisms [40–42]. Future work is required to characterize the interaction between TCP8 and *FLC* in detail. TCP14 and TCP15 have been shown to interact with *MODIFIER OF snc1–1* (*MOS1*), which directly interacts with Suppressor of FRIGIDA 4—a transcriptional activator of *FLC* [28, 43]. In this scenario, TCP8 may also regulate *FLC* mRNA level through interaction with MOS1. Alternatively, TCP8 may compete the interaction between TCP14, TCP15 with MOS1, as protein-protein interactions between TCPs are prevalent [24, 35]. Previous studies have shown that the AP2-domain transcription factors TEMPRANILLO1 (TEM1) and TEM2 inhibit flowering under LD condition through direct transcriptional repression of *FT* [44]. Moreover, TEMs also regulate flowering involving the gibberellin levels and miR172, both required to orchestrate floral transition under SD conditions [45, 46]. Interestingly, we detected up-regulated levels of *TEM1* and *TEM2* in *TCP8* overexpression lines under LD condition (Additional file 7: Figure S7). Examining the putative relationship between TCP8 and TEMs may provide new clues for their functions.

In this study, we found that *TCP8* overexpression inhibited plant growth in a dose-dependent manner (Additional file 1: Figure S1a). The *35S::TCP8* plants showed retarded growth earlier in development but the adult plants established a normal plant height comparable to the wild-type control (Additional file 1: Figure S1b). *TCP8* and other TCP genes are known to participate in cell-cycle control, presumably through regulating *CYCA1;1* and *CYCA2;3* [28, 33, 47]. Thus, the growth inhibition in *35S::TCP8* may be related to the misregulation of cell-cycle. Vernalization could reverse the late-flowering phenotype but not the growth inhibition of *35S::TCP8* plants, suggesting independent regulation of flowering time and cell-cycle by TCP8, and TCP8-related cell-cycle regulation has negligible role in flowering control in our study.

## Conclusions

Our study demonstrates that *TCP8* regulates plant flowering in an FLC-dependent manner. The findings of this study expand our knowledge on the molecular basis of how *TCPs*, especially *TCP8*, control flowering time and provide evidence for the interaction between *TCP8* and *FLC*.

## Methods

### Plant materials and growth conditions

All mutants and transgenic lines used in this study were in the *Arabidopsis thaliana* ecotype Col-0 background. The T-DNA insertion mutant *tcp8–1* (*CS875709*) was obtained from the Arabidopsis Biological Resource Center (ABRC, https://abrc.osu.edu/) and genotyped as previously reported [24]. For transgenic plants, the corresponding plasmid was transformed into WT Col-0 or *flc-6* (*SALK_041126*) by floral dipping and the transgenic plants were selected by half-strength Murashige and Skoog (MS) medium supplemented with proper antibiotic. For *TCP8* overexpression lines, homozygous T3 transgenic plants were used for further analysis. For the co-segregation analysis, *35S::TCP8* or *pTCP8::TCP8* plants crossed with *flc-6*, and the resulting F2 progenies in each population were genotyped. All primers used for genotyping are listed in Additional file 8: Table S1.

Imbibed seeds were sowed and stratified in a cold room at 4 °C for 3 days to break dormancy and then transferred to 22 ± 1 °C at a light intensity of approximately 120 μmol m^− 2^ s^− 1^, with a 16 h light/8 h dark photoperiod for LD and 8 h light/16 h dark photoperiod for SD. The number of rosette leaves was counted after the main stem has bolted to 2 cm. For the vernalization treatment, the imbibed seeds were grown on half-strength MS medium at 4 °C for 4 weeks under dim light and then transferred to 22 ± 1 °C and grown under LD condition.

### Vector construction

A 1582 bp promoter fragment upstream of the *TCP8* translation start site was amplified by PCR using primers pTCP8–1582-S/A, and then cloned into the pCAMBIA1301 (GeneBank accession number AF234297) vector through *Kpn*I/*Bgl*II double digestion to generate the *pTCP8*::GUS construct (the *TCP8* promoter-driven GUS expression construct). For *TCP8* overexpression, the coding sequence (CDS) of *TCP8* was amplified using primers OE-TCP8-S/A and cloned into the pCHF3 vector by *Sac*I/*Bam*HI double digestion to generate the *35S::TCP8* vector. For *pTCP8*::*TCP8* vector construction, a DNA fragment containing the *TCP8* coding region as well as the 1582 bp promoter region was amplified from Col-0 genomic DNA using primers pTCP8-TCP8-S/A and cloned into the pCAMBIA1301 through *Pst*I/*Bstp*I double digestion. To construct the *35S*::*TCP8-EAR* vector, DNA fragment containing the EAR motif was synthesized using primers EAR-S/A and cloned into the *35S::TCP8* vector via *Bam*HI/*Pst*I double digestion. To construct the *pTCP8*::*TCP8-EAR* vector, a 1351 bp promoter fragment upstream of the *TCP8* translation start site was amplified using primers pTCP8–1351-S/A and cloned into the *35S*::*TCP8-EAR* vector through *Eco*RI/*Sac*I double digestion. Primers used for vector construction are summarized in Additional file 8: Table S1.

### Histochemical GUS assays

For the histochemical detection of GUS activity, plant tissues were immersed in the 5-Bromo-4-chloro-3-indolyl β-D-glucuronic acid (X-Gluc) solution (containing 750 mg ml^− 1^ X-Gluc, 0.2 mM K_3_Fe(CN)_6_, 0.2 mM K_4_Fe(CN)_6_ and 0.2% Triton X-100, pH = 7.2) in the vacuum for 15 min at room temperature and incubated at 37 °C overnight. The samples were then washed with 70% ethanol several times until transparent before examined under the microscope.

### RNA extraction and quantitative real-time PCR

To investigate the transcript profile of *TCP8*, different tissues of 40-day-old WT Col-0 plants grown under LD condition were harvested. For detecting the transcript levels of *TCP8* in Col-0 and the *35S::TCP8*, rosette leaves were collected. To detect the transcript levels of *FLC* and genes involved in the autonomous pathways, ten-day-old seedlings grown under LD condition were collected. Total RNA was extracted from the tissues of Col-0 and the transgenic lines using the TRIzol reagent (Invitrogen), DNase I (TaKaRa) was used to wipe out genomic DNA. Two micrograms of total RNA of each sample was used for first-strand cDNA synthesis with M-MLV reverse transcriptase (TaKaRa).

RT-qPCR was performed as previously described [48]. Briefly, reverse-transcribed cDNA was used as the template for RT-qPCR. The RT-qPCR reactions were performed using SYBR Green (TaKaRa) on an iCycler (Bio-Rad) following the manufacturer’s instructions. *ACTIN2* was used as the endogenous control for normalizing the transcript levels of the tested genes. All experiments were performed in three independent biological replicates and three technical replicates. Data are represented as mean ± standard deviation (S.D.). Asterisks indicate significant differences relative to control (*P < 0.05, **P < 0.01, ***P < 0.001, Student’s *t*-test). Primers used for RT-qPCR are listed in Additional file 8: Table S1.

## Supplementary information

**Additional file 1: Figure S1.** *TCP8* overexpression hinders plant growth. **a.** Twenty-day-old wild-type Col-0 and *35S::TCP8* transgenic plants grown in LD condition. **b.** Sixty-day-old wild-type Col-0 and *35S::TCP8* transgenic plants grown in LD condition. (LD: 16 h of light / 8 h of dark).

**Additional file 2: Figure S2.** *pTCP8*::*TCP8* delays flowering. **a.** Relative transcription levels of *TCP8* in different *pTCP8::TCP8* transgenic lines detected by RT-qPCR. Data are represented as mean ± SD of three biological replicates. *ACTIN2* was used as the endogenous control for normalizing the transcription levels of *TCP8*. The transcription level of *TCP8* in Col-0 was arbitrarily set to 1. **b.** Forty-day-old wild-type Col-0 and *pTCP8::TCP8* transgenic plants grown in LD condition. **c.** The number of rosette leaves in wild-type Col-0 and *pTCP8::TCP8* plants before bolting (Student’s *t*-test: *P < 0.05, **P < 0.01, ***P < 0.001).

**Additional file 3: Figure S3.** TCP8 failed to bind the *FLC* promoter in vitro and in vivo. **a.** EMSA detection of TCP8 binding to *FLC* promoter fragment. The *ICS1* promoter containing a TCP binding site was used as a positive control. **b.** TCP8 failed to co-precipitated DNA fragments around *FLC* locus in the chromatin immunoprecipitation (ChIP) assay. The *ICS1* promoter was used as a positive control. The 18S rRNA gene was used to normalize the quantitative PCR results for each of the ChIP samples. Values are means ± SD of three quantitative PCR measurements.

**Additional file 4: Figure S4.** Detection of autonomous pathway genes and *COOLAIR* levels in *TCP8* overexpression plants. **a.** Overexpression of *TCP8* down-regulates a set of autonomous pathway genes expression. Relative transcription levels of autonomous pathway genes in *TCP8* overexpression transgenic lines detected by RT-qPCR. Data are represented as mean ± SD of three biological replicates. *ACTIN2* was used as the endogenous control for normalizing the transcription levels of genes detected. The transcription level of each gene in Col-0 was arbitrarily set to 1. (Student’s *t* test: * *P* < 0.05) **b.** Relative transcription levels of different *COOLAIR* isoforms in *TCP8* overexpression transgenic lines detected by RT-qPCR. Data are represented as mean ± SD of three biological replicates. *ACTIN2* was used as the endogenous control for normalizing the transcription levels of different *COOLAIR* isoforms. The transcription level of *COOLAIR* in Col-0 was arbitrarily set to 1.

**Additional file 5: Figure S5.** The flowering phenotypes of *pTCP8::TCP8* and *flc-6* crossed F2 progenies.

**Additional file 6: Figure S6.** The flowering phenotypes of *tcp8–1* in LD condition. **a.** Forty-day-old plants of different genotypes grown in LD condition. **b.** The number of rosette leaves in Col-0 and *tcp8–1* plants before bolting.

**Additional file 7: Figure S7.** Detection of *TEM1* and *TEM2* expression levels in *TCP8* overexpression plants in LD condition. Forty-day-old plants of different genotypes grown in LD condition. Relative transcription levels of *TEM1* and *TEM2* in *TCP8* overexpression transgenic lines detected by RT-qPCR. Data are represented as mean ± SD of three biological replicates. *ACTIN2* was used as the endogenous control for normalizing the transcription levels of genes detected. The transcription level of each gene in Col-0 was arbitrarily set to 1. (Student’s *t* test: * *P* < 0.05).

**Additional file 8: Table S1.** Primers used in this paper.

## Data Availability

The datasets generated and analyzed during the current study are available from the corresponding author (X. Wang) on reasonable request.

## Abbreviations

35S CaMV: 35S cauliflower mosaic virus
CCA1: CIRCADIAN CLOCK ASSOCIATED 1
ChIP: Chromatin immunoprecipitation
CO: CONSTANS
Col-0: Columbia-0
EMSA: Electrophoretic mobility shift assay
FCA: FLOWERING CONTROL LOCUS A
FLC: FLOWERING LOCUS C
FLD: FLOWERING LOCUS D
FLK: FLOWERING LOCUS KH DOMAIN
FPA: FLOWERING LOCUS PA
FRI: FRIGIDA
FT: FLOWERING LOCUS T
FVE: FLOWERING LOCUS VE
FY: FLOWERING LOCUS Y
GFP: Green fluorescent protein
GUS: β-glucuronidase
LD: LUMINIDEPENDENS
RT-qPCR: Real-time quantitative polymerase chain reaction
SOC1: SUPPRESSOR OF OVEREXPRESSION OF CONSTANS 1
TCP: Teosinte Branched 1 / Cycloidea / Proliferating Cell Factors
TEM1/2: TEMPRANILLO 1/2
