# Supplementary figures and images for: Overexpression of TCP8 delays Arabidopsis flowering through a FLOWERING LOCUS C-dependent pathway
Source: BMC Plant Biol. 2019 Dec 3;19:534. doi: 10.1186/s12870-019-2157-4 (PMC6889539; doi:10.1186/s12870-019-2157-4)

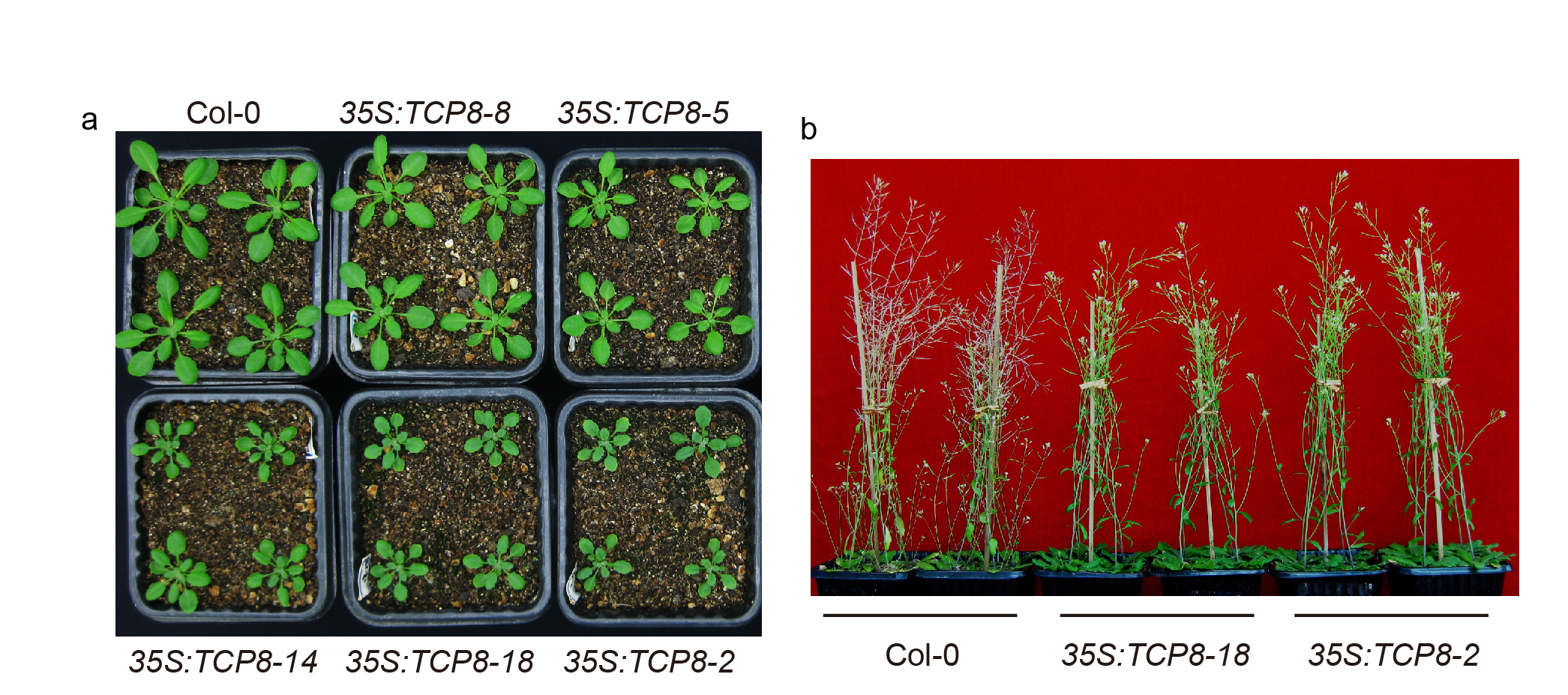

Supplement: Supplementary file 1 — Additional file 1: Figure S1. TCP8 overexpression hinders plant growth. a. Twenty-day-old wild-type Col-0 and 35S::TCP8 transgenic plants grown in LD condition. b. Sixty-day-old wild-type Col-0 and 35S::TCP8 transgenic plants grown in LD condition. (LD: 16 h of light / 8 h of dark). [file 12870_2019_2157_MOESM1_ESM.jpg]

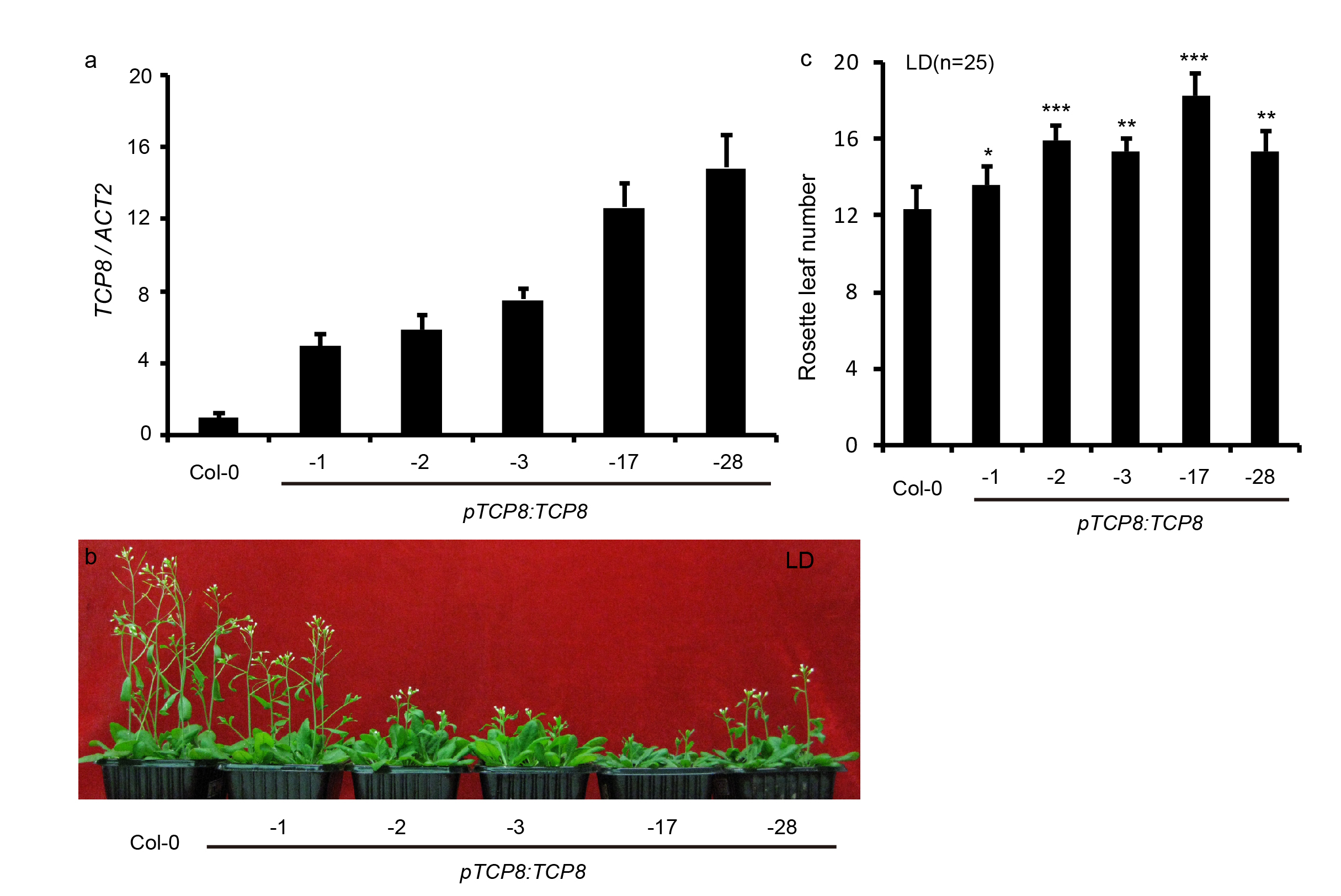

Supplement: Supplementary file 2 — Additional file 2: Figure S2. pTCP8::TCP8 delays flowering. a. Relative transcription levels of TCP8 in different pTCP8::TCP8 transgenic lines detected by RT-qPCR. Data are represented as mean ± SD of three biological replicates. ACTIN2 was used as the endogenous control for normalizing the transcription levels of TCP8. The transcription level of TCP8 in Col-0 was arbitrarily set to 1. b. Forty-day-old wild-type Col-0 and pTCP8::TCP8 transgenic plants grown in LD condition. c. The number of rosette leaves in wild-type Col-0 and pTCP8::TCP8 plants before bolting (Student’s t-test: *P < 0.05, **P < 0.01, ***P < 0.001). [file 12870_2019_2157_MOESM2_ESM.jpg]

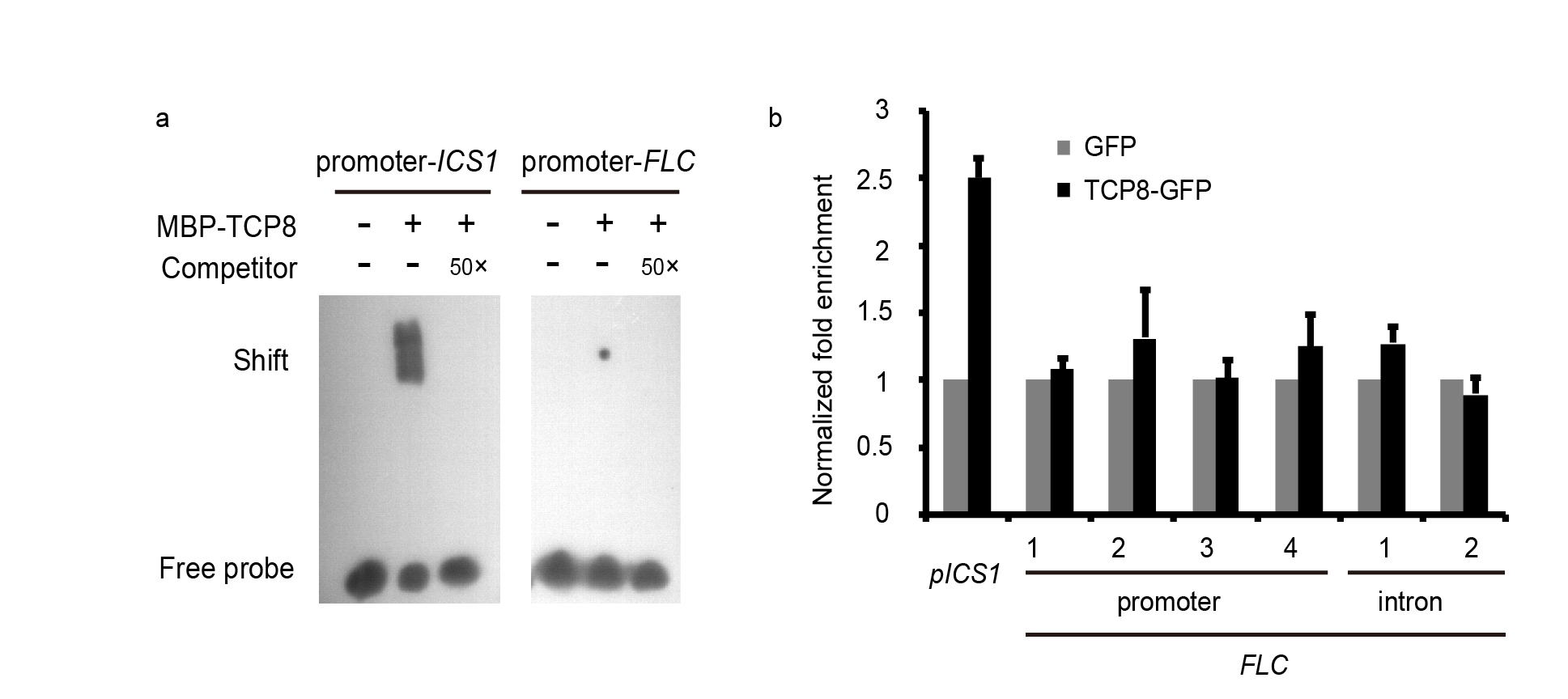

Supplement: Supplementary file 3 — Additional file 3: Figure S3. TCP8 failed to bind the FLC promoter in vitro and in vivo. a. EMSA detection of TCP8 binding to FLC promoter fragment. The ICS1 promoter containing a TCP binding site was used as a positive control. b. TCP8 failed to co-precipitated DNA fragments around FLC locus in the chromatin immunoprecipitation (ChIP) assay. The ICS1 promoter was used as a positive control. The 18S rRNA gene was used to normalize the quantitative PCR results for each of the ChIP samples. Values are means ± SD of three quantitative PCR measurements. [file 12870_2019_2157_MOESM3_ESM.jpg]

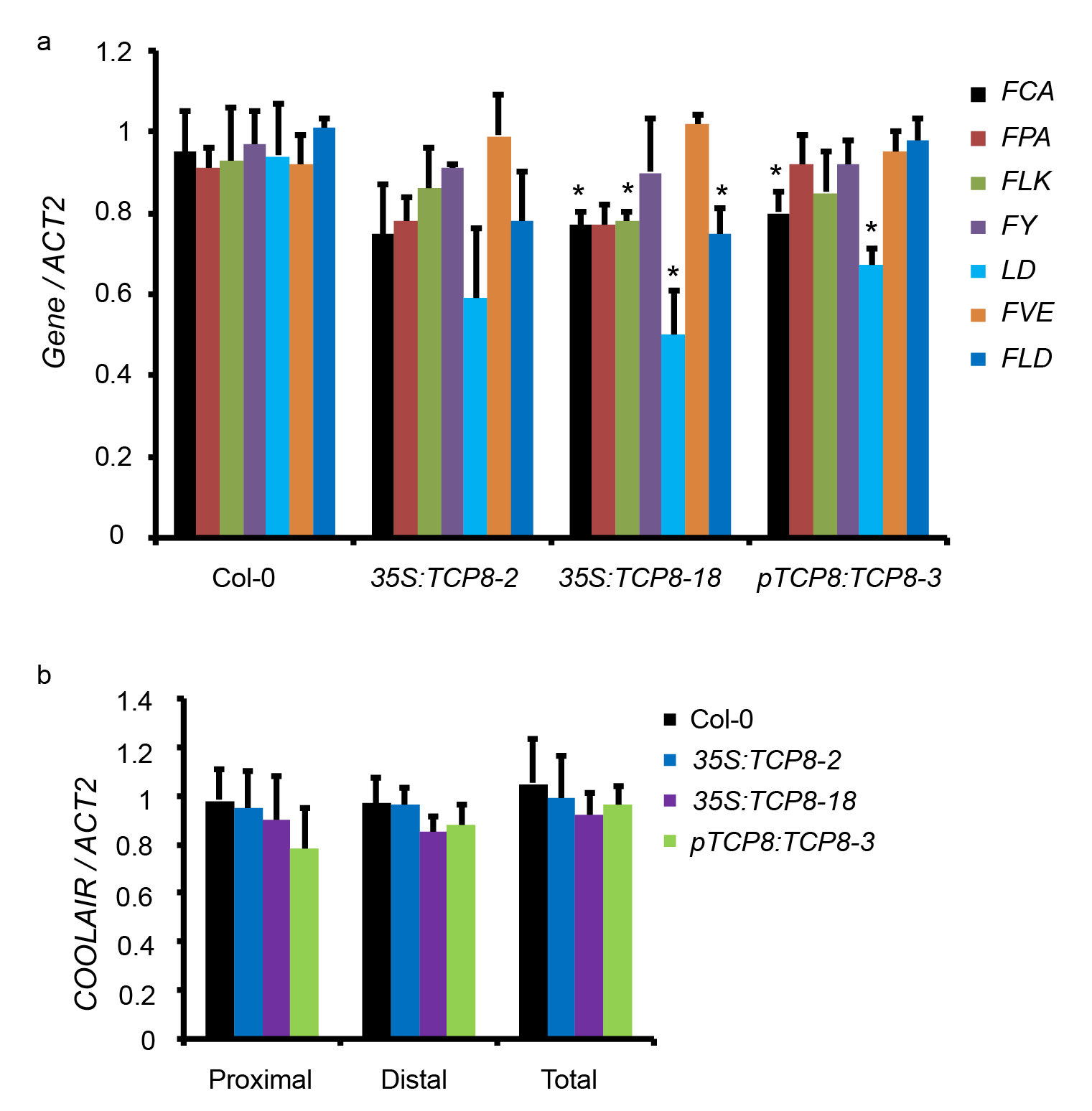

Supplement: Supplementary file 4 — Additional file 4: Figure S4. Detection of autonomous pathway genes and COOLAIR levels in TCP8 overexpression plants. a. Overexpression of TCP8 down-regulates a set of autonomous pathway genes expression. Relative transcription levels of autonomous pathway genes in TCP8 overexpression transgenic lines detected by RT-qPCR. Data are represented as mean ± SD of three biological replicates. ACTIN2 was used as the endogenous control for normalizing the transcription levels of genes detected. The transcription level of each gene in Col-0 was arbitrarily set to 1. (Student’s t test: * P < 0.05) b. Relative transcription levels of different COOLAIR isoforms in TCP8 overexpression transgenic lines detected by RT-qPCR. Data are represented as mean ± SD of three biological replicates. ACTIN2 was used as the endogenous control for normalizing the transcription levels of different COOLAIR isoforms. The transcription level of COOLAIR in Col-0 was arbitrarily set to 1. [file 12870_2019_2157_MOESM4_ESM.jpg]

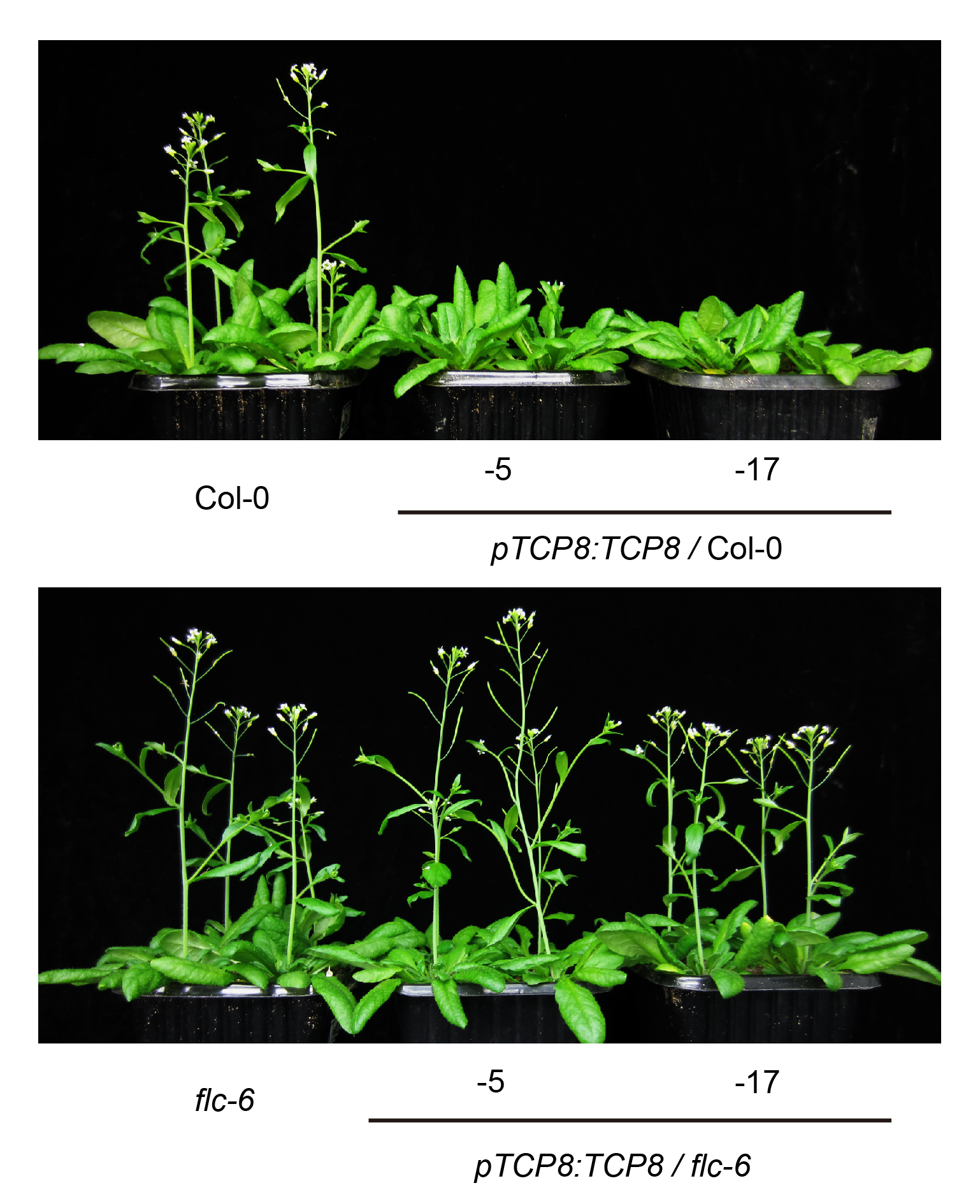

Supplement: Supplementary file 5 — Additional file 5: Figure S5. The flowering phenotypes of pTCP8::TCP8 and flc-6 crossed F2 progenies. [file 12870_2019_2157_MOESM5_ESM.jpg]

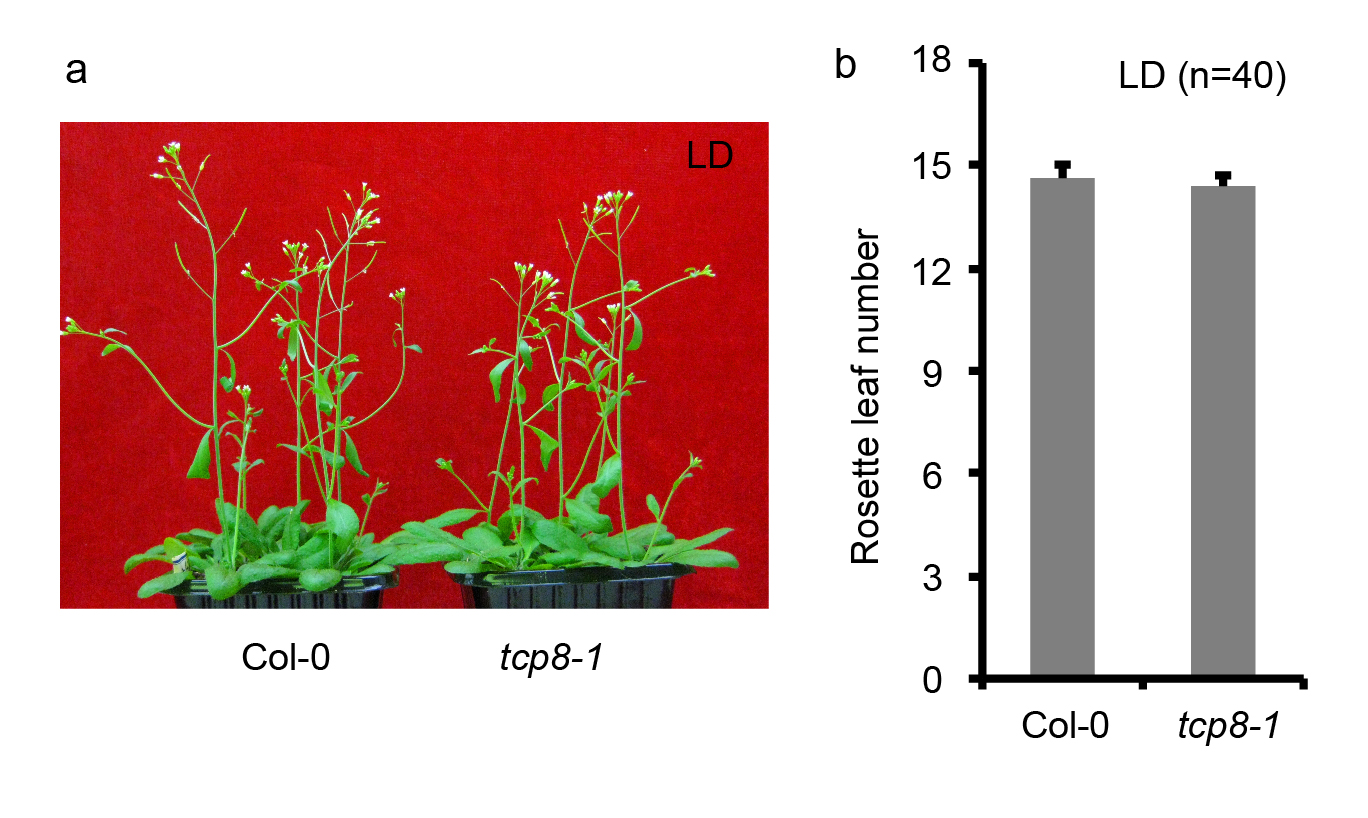

Supplement: Supplementary file 6 — Additional file 6: Figure S6. The flowering phenotypes of tcp8–1 in LD condition. a. Forty-day-old plants of different genotypes grown in LD condition. b. The number of rosette leaves in Col-0 and tcp8–1 plants before bolting. [file 12870_2019_2157_MOESM6_ESM.jpg]

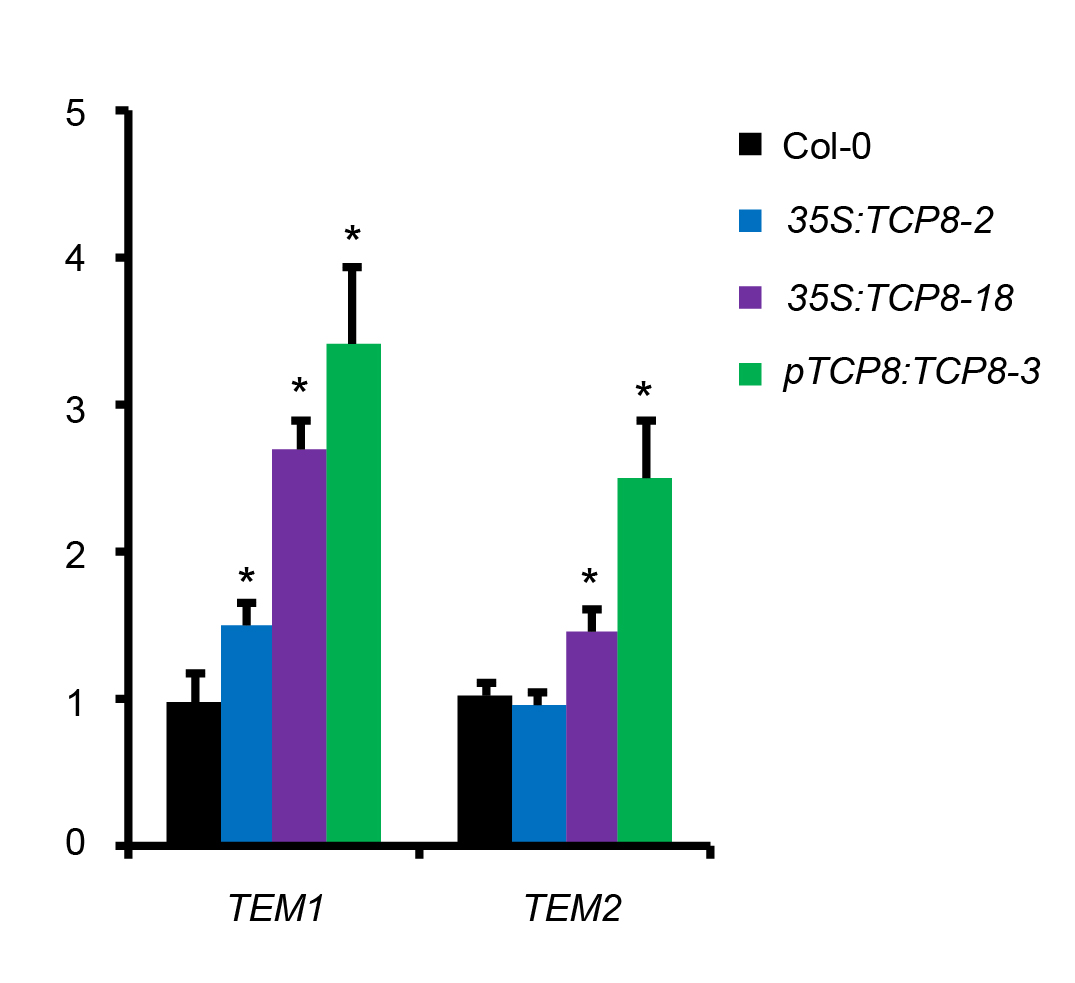

Supplement: Supplementary file 7 — Additional file 7: Figure S7. Detection of TEM1 and TEM2 expression levels in TCP8 overexpression plants in LD condition. Forty-day-old plants of different genotypes grown in LD condition. Relative transcription levels of TEM1 and TEM2 in TCP8 overexpression transgenic lines detected by RT-qPCR. Data are represented as mean ± SD of three biological replicates. ACTIN2 was used as the endogenous control for normalizing the transcription levels of genes detected. The transcription level of each gene in Col-0 was arbitrarily set to 1. (Student’s t test: * P < 0.05). [file 12870_2019_2157_MOESM7_ESM.jpg]
